# Influence of Electric, Magnetic, and Electromagnetic Fields on the Circadian System: Current Stage of Knowledge

**DOI:** 10.1155/2014/169459

**Published:** 2014-07-22

**Authors:** Bogdan Lewczuk, Grzegorz Redlarski, Arkadiusz Żak, Natalia Ziółkowska, Barbara Przybylska-Gornowicz, Marek Krawczuk

**Affiliations:** ^1^Department of Histology and Embryology, Faculty of Veterinary Medicine, University of Warmia and Mazury, Oczapowskiego Street 13, 10-719 Olsztyn, Poland; ^2^Department of Mechatronics and High Voltage Engineering, Gdansk University of Technology, Własna Strzecha Street 18A, 80-233 Gdansk, Poland; ^3^Department of Electrical Engineering, Power Engineering, Electronics, and Control Engineering, University of Warmia and Mazury, Oczapowskiego Street 11, 10-736 Olsztyn, Poland

## Abstract

One of the side effects of each electrical device work is the electromagnetic field generated near its workplace. All organisms, including humans, are exposed daily to the influence of different types of this field, characterized by various physical parameters. Therefore, it is important to accurately determine the effects of an electromagnetic field on the physiological and pathological processes occurring in cells, tissues, and organs. Numerous epidemiological and experimental data suggest that the extremely low frequency magnetic field generated by electrical transmission lines and electrically powered devices and the high frequencies electromagnetic radiation emitted by electronic devices have a potentially negative impact on the circadian system. On the other hand, several studies have found no influence of these fields on chronobiological parameters. According to the current state of knowledge, some previously proposed hypotheses, including one concerning the key role of melatonin secretion disruption in pathogenesis of electromagnetic field induced diseases, need to be revised. This paper reviews the data on the effect of electric, magnetic, and electromagnetic fields on melatonin and cortisol rhythms—two major markers of the circadian system as well as on sleep. It also provides the basic information about the nature, classification, parameters, and sources of these fields.

## 1. Introduction

One of the side effects of each electrical device work is the electromagnetic field generated near its workplace. All organisms, including humans, are exposed daily to the influence of different types of this field, characterized by distinct physical parameters. Therefore, it is important to accurately determine the effects of electromagnetic field on organisms. All electrically powered devices and transmission lines generate the low frequency (usually 50 or 60 Hz) field, which has a quasi-stationary character and its two components—the electric and magnetic field—can be analysed separately. This field is considered as having a potentially negative impact on organisms, although the mechanism of its biological action remains unknown. On the other hand, electronic devices, such as mobile phones, television sets or radio transmitters, emit electromagnetic radiation with high frequencies (from 300 MHz to 300 GHz). High energy radiation of this type causes a thermal effect that may increase the temperature of tissues and organs and also cause serious damage to cells. The international agency for research on cancer (IARC) in 2002 classified the extremely low frequency magnetic field generated by electrical devices as possibly carcinogenic to humans [1]. In 2011, the radio frequencies of electromagnetic fields were qualified by IARC and WHO as possibly increasing the risk of malignant brain tumour development [2].

The visible part of electromagnetic radiation, with a relatively narrow frequency band from 389 to 789 THz, plays a key role in the regulation of the diurnal rhythms by having influence on the activity of the suprachiasmatic nucleus via melanopsin-positive ganglion cells of the retina [3]. However, several reports have provided evidence that electric and magnetic fields also influence the circadian system. It has been suggested that a deficiency in melatonin secretion may be responsible for the oncogenic action of the electromagnetic field [4].

The aim of the paper was to review the data on the effects of electric, magnetic, and electromagnetic fields on melatonin and cortisol rhythms, two major markers of the circadian system as well as on sleep. We also included information on the nature, physical parameters, classification, and sources of fields, which may be useful for biologists and medical doctors.

## 2. Nature of Electric, Magnetic, and Electromagnetic Forces

In physical sciences, the electromagnetic field is the state of space characterised by electrodynamic nature of forces acting on electrically charged objects. In that context, the electromagnetic field can be thought of as consisting of two independent components [5]:

- electric—represented by a state of space, known as an electric field, in which Coulomb forces act on stationary electrically charged objects,
- magnetic—represented by a state of space, known as a magnetic field, in which Lorenz forces act on nonstationary (moving) electrically charged objects (representing electric currents).

It may be interesting to note that according to the special theory of relativity, electric and magnetic fields are two aspects of the same phenomenon depending on a chosen reference frame of observation—an electrical field in one reference frame may be perceived as a magnetic field in a different reference frame.

Within the range of their influence, the electromagnetic fields may affect physical objects, including living organisms. The effects of this influence depend on many factors. Among these, the most important are [5]

- field intensity—in the case of the electric field, its intensity *E* is expressed in volts per metre (V/m), while in the case of the magnetic field (MF) its intensity *H* is expressed in amperes per metre (A/m),
- distance *R* from an object expressed in metres (m),
- frequency *f* of radiated energy—in the case of time dependent fields it is expressed in hertz (Hz), while for time independent fields their frequency *f* equals 0,
- surface power density *P* (specific power) representing the intensity of radiated energy (power) with the area throughout this energy being radiated, expressed in watts per square metre (W/m^2^).

It is worth mentioning at this point that the intensity of a magnetic field *H* is expressed in amperes per metre (A/m) according to the SI standards. However, in the literature and scientific practice, very often, the induction of a magnetic field *B* is used instead, which is expressed in tesla (T). These quantities—*H* and *B*—are interrelated through the medium magnetic permeability *μ*.

## 3. Electromagnetic Fields in the Habitat of Living Organisms

Electromagnetic radiation and fields have been accompanying living organisms since the dawn of life on Earth. However, their current intensity and omnipresence should be attributed, first of all, to human activity—technological advances in modern engineering related to the development and practical use of electrical power transmission systems, electrical equipment, and telecommunications.

**Figure 1:**
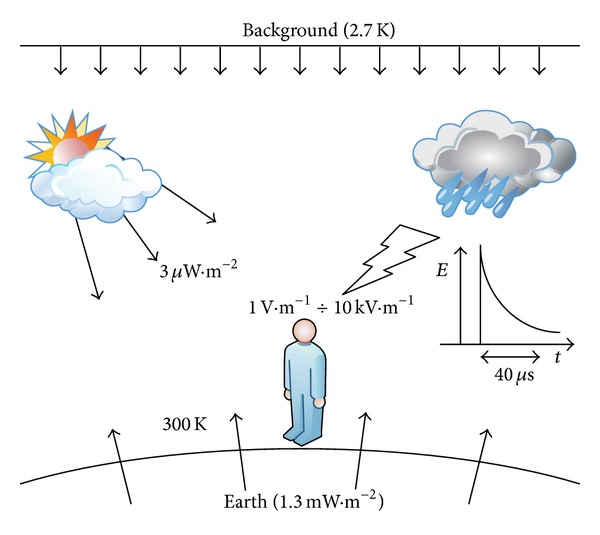
Natural radiation sources present on Earth (based on [6]).

The sources of electromagnetic radiation and fields can be divided into natural and nonnatural ones. The natural sources include celestial bodies such as stars and magnetars, Earth and biological processes involving the flow of electrical impulses in living organisms (Figure 1). The electromagnetic radiation that reaches the Earth's surface from space as microwave background radiation is a consequence of the big bang and the evolution of the universe in the very first seconds of its existence. This type of radiation is characterised by its thermal energy distribution as the most perfect black body in nature and has a nearly ideal Planck spectrum at a temperature around 2.7 K, while the maximum of its surface power density corresponds to the wavelength of 272 GHz [6]. The solar radiation that reaches the Earth's surface has relatively small surface power density around 3 *μ*W/m^2^ [6] and comprised of distinctive frequency bands, so-called atmospheric windows, representing those frequency bands that are not absorbed by the Earth atmosphere. They can be listed as

- radio window—represented by electromagnetic wavelengths starting from 15 MHz up to 300 GHz,
- optical window—represented by electromagnetic wavelengths starting from 150 THz up to 1000 THz,
- microwave window—represented by electromagnetic wavelengths starting from 23.1 THz up to 37.5 THz.

The magnetic field of Earth is another natural field originating from the planet core that extends to a vast space surrounding Earth, known as the magnetosphere. An important source of strong electromagnetic fields is atmospheric discharges, known as lightning. Rapid radiation releases, which accompany these natural phenomena, are characterised by high power densities and high frequencies. In living organisms, electromagnetic fields originate from the transmission of signals in the nervous system and from structures autonomously generating electrical impulses (like the heart).

The history of nonnatural sources of electromagnetic radiation and fields is relatively short and covers only the last hundred years. Nonnatural sources of electromagnetic radiation or fields are attributed to two groups. The first group includes ionising radiation, characterised by a relatively high energy that may result in the ionisation of matter particles. The presence of this kind of radiation has primarily natural reasons (the statistical annual exposure dose is around 2.4 mSv). However, nonnatural sources of ionising radiation, such as technical devices, in which various radioactive isotopes are used, are currently considered to be the most important problems in public health protection. The second group comprises nonionising radiation of energy, which is too low to ionise matter particles. The common sources of this kind of radiation are all means used for electrical power production, transmission, and utilisation (high-voltage power lines, substations, motors, generators, industrial and domestic appliances, home wiring, etc.). Very important sources of electromagnetic radiation include telecommunication systems (radio, television, internet, and Wi-Fi) as well as medical devices used for diagnosis or therapy.

According to the European Commission, nonionizing radiation can be divided into several levels [7]:

- static fields,
- extremely low frequency fields (ELF fields),
- intermediate frequency fields (IF fields),
- radio frequency fields (RF fields).

In order to illustrate the authors' considerations, typical sources of electromagnetic fields/radiation influencing living organisms and mentioned above are listed and described in Table 1.

## 4. Effects of Electric, Magnetic, and Electromagnetic Fields on the Diurnal Rhythm of Melatonin Secretion

Melatonin is the main hormone of the circadian timing system in all vertebrates including the human [8]. The diurnal rhythm of its secretion in the mammalian pineal gland is driven by the suprachiasmatic nucleus—the central endogenous oscillator, directly connected with the retina [8–10]. Under physiological condition, the regulatory mechanisms ensure that this rhythm is properly entrained to the light-dark cycle and, therefore, the elevated night-time melatonin secretion can serve for all cells of the body as a clock and a calendar [8, 11, 12]. Melatonin plays a key role in the control of many physiological processes occurring in daily or seasonal rhythms, like sleep, metabolism, and reproduction [13]. Moreover, melatonin is also involved in the regulation of immune system [14], cardiovascular system [15], and cancer development [13, 16, 17]. It is also a very potent free radical scavenger [18].

It is worth to note that the level of melatonin secretion differs markedly between individuals, in both humans [19, 20] and animals [21, 22]. Based on urinary melatonin measurements, the human population could be divided into low and high melatonin excretors [19, 20]. The study on the sheep demonstrated that interindividual variability in a plasma melatonin level is under strong genetic control and it is related to the pineal gland weight and melatonin secretion, but not to the hormone catabolism [21]. The individual diurnal profiles of plasma melatonin are highly repeatable on consecutive days, weeks, and months, in both humans and animals [20, 22]. The level of nocturnal melatonin secretion decreases with age [23].

Several factors, like light pollution during night or moving across time zones, may lead to the disruption of the melatonin secretion rhythm and circadian disorganization, which undoubtedly has a negative impact on various aspects of health [13, 14, 16, 24, 25].

The melatonin secretion by the pineal gland is generally regarded as particularly sensitive to electric, magnetic, and electromagnetic field influences. The effects of these fields on pineal activity have been analyzed in epidemiological studies [26–41] and experimental investigations carried out using different* in vivo* [42–94] and* in vitro* models [95–100].

### 4.1. Epidemiological Studies

The epidemiological studies provided interesting and very important data on the influence of electromagnetic fields on melatonin and its metabolite—6-sulfatoxymelatonin—in humans. Many of these investigations concerned the effects of an extremely low frequency magnetic field (ELF-MF), which is generated by outdoor high- and medium-voltage electricity power lines, indoor electrical power supply, and electrical appliances [25].

The relations between exposure to the magnetic fields with a frequency of 16.7 Hz and human health have been intensively studied in railway workers [26, 101, 102]. Pfluger and Minder [26] compared, using a repeated measures design, the urinary excretion of 6-sulfatoxymelatonin in 108 male Swiss railway workers between leisure periods and days following the start of service on electrically powered engines or doing other tasks. The study demonstrated that the urinary excretion of 6-sulfatoxymelatonin was lower on work days than leisure days among engine drivers exposed to a 16.7 Hz magnetic field with an average strength of 20 *μ*T, but not among other workers. It should be noted that epidemiological studies of Swiss railway workers demonstrated significantly increased (0.9% per *μ*T-year of cumulative exposure) leukemia mortality [101]. The statistical data also suggest a link between occupational exposition to a magnetic field with a frequency of 16.7 Hz and the risk of Alzheimer's disease [102].

Humans are widely exposed to magnetic fields with a frequency of 50 Hz (in Europe) or 60 Hz (in North America) generated by the electrical power supply and electrical devices, commonly used in homes and workplaces. The decreased excretion of 6-sulfatoxymelatonin in urine was observed in electrical utility workers, who were exposed to magnetic fields with a frequency of 60 Hz [27–29]. Significant changes were noted after the second day of the working week and the effect of the magnetic field exposition was the most prominent in subjects with low workplace light exposures [28]. Further, it was demonstrated that a decrease in excretion of 6-sulfatoxymelatonin occurred in workers exposed for more than two hours and in a 3-phase environment [29]. No change was found in people working in a 1-phase environment. A weak effect of occupational exposure to low-intensity magnetic field on 6-sulfatoxymelatonin excretion was also observed in female workers [30].

Davis et al. [31] suggested that domestic exposure to a 60 Hz magnetic field decreased pineal activity in women, primarily those using medications. The level of 6-sulfatoxymelatonin excretion was lower in infants kept in incubators and rose when they were moved to a place free from electrical devices [103]. The analysis performed by Juutilainen and Kumlin [32] suggests that exposure to a magnetic field with a frequency of 50 Hz may enhance the effects of night-time light exposure on melatonin production; however, the study was performed on a relatively small group of subjects.

It should be underlined that a moderate number of epidemiological studies showed no effect of the exposure to ELF-MF on melatonin secretion [33–37]. Gobba et al. [33] noted similar levels of 6-sulfatoxymelatonin excretion in two groups of workers exposed to fields ≤0.2 *μ*T and >0.2 *μ*T. No association between residential exposure to a 60 Hz magnetic field and 6-sulfatoxymelatonin excretion was observed in adults aged 50–81 years [34]. Touitou et al. [35] showed that the long-term exposure to ELF-MF did not change the level and diurnal secretion of melatonin. These data suggest that magnetic fields do not have cumulative effects on melatonin secretion in humans.

In contrast to ELF-MF, much less attention has been paid in epidemiological studies to the effects of intermediate frequency range (300 Hz to <10 MHz) and radio frequency range (10 MHz to 300 GHz) electromagnetic fields. No changes in urinary 6-sulfatoxymelatonin excretion were found in women residing near radio and television broadcasting transmitters [38]. The use of a mobile phone for more than 25 minutes a day decreased the level of melatonin secretion [39]. Broadcast transmitters with short-wave electromagnetic fields (6–22 MHz) reduced melatonin secretion by 10% [40]. A study carried out on 50 electronic equipment service technicians, exposed to different kinds of fields, found significantly decreased levels of serum melatonin compared to the control group [41].

### 4.2. Experimental Studies on Volunteers

In contrast to the epidemiological studies, the majority of investigations performed on volunteers found no effect of ELF-MF on melatonin or/and 6-sulfatoxymelatonin levels [42–51]. In a study by Warman et al. [42], 2-hour-long exposure to a 50 Hz field at an intensity of 200–300 *μ*T did not induce significant changes in the nocturnal melatonin rise. Similarly, the exposure of volunteers for one night to 50 Hz field at an intensity of 20 *μ*T had no effect on plasma melatonin level [43]. Selmaoui et al. [44] demonstrated that nocturnal acute exposure to either continuous or intermittent 50 Hz linearly polarized magnetic fields of 10 *μ*T does not affect melatonin secretion in humans. In a series of experiments performed by Graham et al. [45–49], the nocturnal secretion and metabolism of melatonin were not altered in humans by the exposure to ELF-MF at intensities within the occupational-exposure range for one or more nights. No changes in salivary melatonin were found after exposing volunteers to a 16.7 Hz electromagnetic field [50, 51]. In contrast to the data presented above, Davis et al. [52] demonstrated that the exposure to a magnetic field of 0.5 to 1 *μ*T greater than the ambient levels for 5 consecutive nights reduced the excretion of 6-sulfatoxymelatonin in women.

### 4.3. Experimental Studies on Animals

The majority of* in vivo* experiments concerning the influence of magnetic field exposure on pineal activity have been conducted on laboratory rodents [53–85].

Highly variable results were obtained in the studies on the effects of ELF-MF. The continuous exposition of Sprague-Dawley rats to a 10 *μ*T 50 Hz magnetic field for 91 days decreased the blood melatonin level [53]. However, another study from the same group failed to demonstrate a consistent effect of a 100 *μ*T 50 Hz magnetic field exposure on melatonin levels in rats, as a decline or no changes were observed [54]. A decrease in the pineal activity in response to ELF-MF was also noted in several other experiments performed on laboratory rats [55–63] and Djungarian hamsters [64, 65]. On the other hand, an increased excretion of 6-sulfatoxymelatonin was observed in Sprague-Dawley rats exposed to a magnetic field with a frequency of 50 Hz and an intensity of 100 *μ*T for 24 hours [66]. Similarly, Dyche et al. [67] demonstrated that male rats, exposed to the 100 *μ*T magnetic field for 1 month, have a slightly elevated excretion of 6-sulfatoxymelatonin. Increased melatonin secretion after exposure to a weak magnetic field was also reported in the Djungarian hamster by Niehaus et al. [68]. In other studies performed on rats and hamsters, no changes in melatonin secretion were observed in response to a magnetic field with a frequency of 50/60 Hz [69–77]. The lack of influence of ELF-MF on pineal activity was also reported for mice [78].

Studies on rodents have provided interesting data concerning the effect of radio frequency range of electromagnetic field on pineal activity. The exposure of rats to an electromagnetic field of 900 MHz frequency and a specific adsorption of 0.9 W*·*kg^−1^ (mobile phone) lasting 2 hours a day and repeated for 45 days resulted in a statistically significant decrease in pineal melatonin content [81]. Moreover, a field of 1800 MHz frequency and a power of 200 W*·*cm^−2^ (2 hours per day for 32 days; 0.5762 W*·*kg^−1^) disturbed the rhythm of melatonin secretion in rats [82]. However, in another experiment, the animals were subjected to a similar field for 30 minutes a day, 5 days a week for 4 weeks and no changes in the level of melatonin in rat serum were noted [83]. Similarly, the exposure of Djungarian hamsters to an electromagnetic field with frequencies of 383, 900, and 1800 MHz (80 m W*·*kg^−1^) for 60 days (24 hours a day) did not result in alternations of the melatonin secretion [84].

Studies on the effects of electric and magnetic fields on nonrodent species have been conducted only occasionally [86–94]. The exposure of dairy cattle to a vertical electric field of 10 kV/m and a uniform horizontal magnetic field of 30 *μ*T for 28 days did not change the nocturnal blood melatonin level [86]. Similarly, no changes in melatonin secretion were observed in other experiments performed on dairy cows [87, 88] and on lambs [89, 90]. The studies of American kestrels reveled that a long-term exposure to electromagnetic fields (60 Hz, 30 *μ*T, 10 kV*·*m^−1^) caused changes in melatonin secretion [91]. The magnetic field increased the level of melatonin in the pineal gland and blood serum of trout during the night [92].

### 4.4. *In Vitro* Studies

*In vitro* studies concerning the effect of electromagnetic fields on melatonin secretion were conducted on the pineal glands of Djungarian hamsters [95, 100] and rats [96–99]. The results of experiments with hamster pineals in the superfusion organ culture demonstrated that ELF-MF with an intensity of 86 *μ*T and a frequency of 16.67 or 50 Hz caused a decrease in melatonin secretion, activated by isoproterenol [95]. A reduction in isoproterenol-stimulated melatonin secretion and activity of arylalkylamine N-acetyltransferase has also been found in studies of rat pinealocytes after exposure to ELF-MF [96, 97]. On the contrary, Lewy et al. [98] noted increased activity of melatonin-synthetizing enzymes, while Tripp et al. [99] found no changes in melatonin secretion in rat pinealocytes in response to ELF-MF.

The effect of exposure to an electromagnetic field with a frequency of 1800 MHz on melatonin secretion from the Djungarian hamster pineal gland was investigated [100] in the same experimental setup which had been used in experiments with ELF-MF [95]. This study demonstrated that both continuous and pulse signals at a specific adsorption level of 800 mW*·*kg^−1^, lasting seven hours, increased the level of isoproterenol-stimulated melatonin secretion [100].

## 5. Effects of Electric, Magnetic, and Electromagnetic Fields on the Diurnal Rhythm of Cortisol Secretion

Cortisol is an essential steroid hormone produced by the adrenal gland. Like melatonin, it exhibits a constant and reproducible diurnal rhythm under physiological conditions [104–107]. Debono et al. [105] in a study of 33 healthy individuals with 20-minute-interval cortisol profiling over 24 hours showed that the cortisol concentration reached the lowest levels at around midnight. It then started to rise at 02:00–03:00 and the peak occurred at around 08:30. Next, the cortisol level slowly decreased back to the nadir. The peak cortisol level in the human blood was approximately 399 nmol/L, while the nadir cortisol level was <50 nmol/L. Like many other physiological processes in the body occurring in daily cycles, the rhythm of cortisol secretion is regulated by the suprachiasmatic nucleus, located in the hypothalamus.

Cortisol governs hunger and appetite, stress, inflammatory response, and many other functions [108–110]. The importance of cortisol is especially evident when it becomes deficient in a state known as adrenal insufficiency [111]. It has been suggested that cortisol acts as a secondary messenger between central and peripheral clocks and may be an important factor in the synchronization of body circadian rhythms [111]. Alterations in the rhythmic production and level of the cortisol lead to significant adverse effects [108, 112]. Children with autism frequently show a large variation in day-time patterns of cortisol and significant elevations in salivary cortisol in response to a nonsocial stressor [113].

Both people and animals live in environments with electromagnetic fields of different origins. They are exposed to electromagnetic field of natural origin, like the magnetic force of Earth and artificial origins, which results from human activities. Variations in the Earth's magnetic field are consequential to all living beings of the planet. In addition, electric and magnetic fields, which exist wherever electricity is generated or transmitted, seem to be very important to exposed organisms.

### 5.1. Experimental Studies on Animals

The results of studies on the effects of electromagnetic field on the secretion of cortisol in animals are very diverse. In Guinea pigs, ELF-MF caused changes in cortisol levels, which depended on the field frequency and intensity [114]. Exposure of animals for 2 h and 4 h per day, over a period of 5 days, to a field of 50 Hz and 0.207 *μ*T showed a significant decrease in cortisol levels [114]. However, in the groups subjected to a field of 5 Hz and 0.013 *μ*T, no significant changes in cortisol were observed after 2 h or 4 h of exposure [114]. In Swiss mice continuously exposed to a low frequency (50 Hz) field for 350 days, a decrease in cortisol value was observed on day 190 of the experiment [115]. No significant differences were noted on days 90 and 350 of the exposure [115]. An increase in the cortisol level was observed in rats exposed to uniform magnetic fields of 10^−3^ T and 10^−2^ T, 1 hour each day for a period of ten days [116]. The exposure of female hamsters to mobile phones working at 950 MHz for short (10 days, 3 h daily) and long (60 days, 3 h daily) periods caused a significant increase in cortisol in comparison with the control group [117].

A lack of electromagnetic field effect on cortisol concentration was also reported. Burchard et al. [118] showed no variation in cortisol concentration, which could be attributed to the exposure of dairy cows to electric and magnetic fields (vertical electric field 10 kV and horizontal magnetic field of 30 mT). In ewe lambs, no effect of the exposition to a 60 Hz magnetic field for 43 weeks on serum cortisol was also reported [119]. A lack of electromagnetic field effect on corticosterone concentration, irrespective of the exposure characteristics and period, was also found in experiments on rats [120, 121].

### 5.2. Studies in Humans

The studies concerning the influence of the Earth's magnetic force on the human body demonstrated that the serum cortisol values were dependent on the direction of the head during sleep in relation to the North and South Magnetic Poles [122]. The biological effect of exposure to man-made electromagnetic fields on humans was the subject of several studies [123–127]. Dentistry is one of the job categories with high exposure to elevated levels of ELF-MF. Exposure of dentists to the fields emitted by cavitrons caused a decrease in the serum cortisol level in comparison with a control group [123]. Low frequency magnetic fields are applied in physiotherapy (magnetotherapy and magnetostimulation). Studies of the long-term application of these procedures suggest a regulating influence of magnetic fields on cortisol concentration [124]. However, it should be stressed that numerous studies found no effect of the magnetic fields 50/60 Hz (1–20 *μ*T) and the radio frequency electromagnetic fields on a level of cortisol, irrespective of the experiment time, age, or sex of individuals or sampling time [125–127].

## 6. Effects of Electric, Magnetic, and Electromagnetic Fields on Sleep

The diurnal rhythms are generated by an internal biological clock system that is synchronized to a 24-hour day by environmental factors, primarily the light-dark cycle. Many rhythms are overt and easy to recognize, such as the sleep-wake cycle, locomotor activity, and feeding behavior.

The sleep-wake cycle is likely the primary output rhythm of the circadian clock, because the regulation of many behavior and physiological activities depends on whether the organism is asleep or awake. Sleep disorders—frequently occurring clinical symptoms—have been hypothesized to be partially related to electromagnetic field exposure. In recent years, there has been an increasing amount of experimental and epidemiological data on the influence of nonionizing electromagnetic fields on brain physiology and sleep [40, 128–144].

Sleep is an endogenous, self-sustained cerebral process. It is possible to measure defined and distinguishable phases of sleep. The low frequency activity (<10 Hz) and the sleep spindle frequency activity (approximately 12–15 Hz) are two silent features of nonrapid eye movement (NREM) sleep that can be quantified and used as markers of sleep regulating processes [145]. Several experiments have shown that electroencephalographic (EEG) spectral power in the alpha (8–12 Hz) and spindle (12–14 Hz) frequencies is enhanced both during and following pulsed-modulated radio frequency field exposure [128–133]. Recently, an increase in delta power (<4.5 Hz) has also been observed [129]. Mann and Röschke [134] reported a reduction of rapid eye movement (REM) sleep and changes in spectral power of EEG during REM sleep in response to a high frequency electromagnetic field emitted by digital mobile radio telephones. Regel et al. [130] performed a study on the influence of radio frequency electromagnetic field exposure by varying the signal intensity in three experimental sessions. The analysis of the sleep EEG revealed a dose-dependent increase of power in the spindle frequency range in NREM sleep. This provided the first indications of a dose-dependent relation between the field intensity and its effect on brain physiology. Huber et al. [137] also demonstrated a power increase in the fast spindle frequency range of EEG during pulse-modulating radio frequency field exposure but not in a dose-dependent manner. It should be also stressed that many studies [135, 139–141] failed to show any effects of the radio frequency field exposure on sleep or sleep EEG.

Despite several reports showing an influence of pulsed-modulated radio frequency electromagnetic field on sleep EEG, the mechanism behind these exposure-induced changes is still unclear. Additionally, there is no supporting evidence that this effect is related to health consequences such as alterations in sleep quality [128–130, 136].

To date, there have been few controlled laboratory studies on sleep EEG under low frequency electric and magnetic fields. Åkerstedt et al. [143] carried out a double-blind, placebo-controlled study on 18 healthy subjects to examine the effects of a 50 Hz magnetic field on sleep. The results showed that sleep efficiency, slow wave sleep, and slow activity as well as subjective depth of sleep were significantly reduced under ELF-MF exposure. Although these results suggest an interference of the low frequency field, the authors emphasize that these alterations are still within a normal range. In a double-blind laboratory study, Graham et al. [144] investigated the effect of a 60 Hz magnetic field on sleep during continuous, intermittent, or sham exposures. They demonstrated that intermittent exposure resulted in clear distortion of sleep and altered sleep architecture compared to sham conditions and continuous exposure. It should be emphasized that field strengths in both cited studies [143, 144] were below those used for medical diagnostic purposes such as magnetic resonance imaging.

The analysis of epidemiological data concerning the sleep quality and melatonin cycle, collected during ten years in the area surrounding a short-wave (6–22 MHz) broadcasting station, provided the evidence that electromagnetic field exposure only affects poor sleepers and that might be a group of people who are sensitive to such exposure [40]. This phenomenon has been described as electromagnetic hypersensitivity, EHS. It was also observed in several other reports [146, 147].

Although a biological explanation for an association between exposure to radio frequency electromagnetic field and impaired sleep quality has not been identified, it is hypothesized that the suppression of night-time melatonin secretion may be involved in this process [148]. Two relatively recent studies suggest an association between the decreased secretion of melatonin during the night and increasing use of mobile phones emitting a radio frequency field [39, 149]. However, four cross-over trials [127, 141, 150, 151] have found no correlation between the exposure to mobile phone handset and the melatonin secretion. The hypothesis of an association between melatonin cycle and electromagnetic field exposure requires further investigation [152].

## 7. Conclusions

The results of studies on the effects of electric, magnetic, and electromagnetic fields on melatonin and cortisol secretion as well as on sleep are largely contradictory. The adverse data related to the influence of these physical factors on secretion of both “circadian” hormones were obtained in all groups of investigations including the epidemiological studies, the studies on volunteers, and the studies on animals. Moreover,* in vitro* investigations on rodent pineals have also brought inconsistent results. The sources of discrepancies remain unknown; however such factors as an inappropriate estimation of exposure level, interferences with other factors like light and medication, differences in a phase of the circadian rhythm during exposure, and interindividual variability in the sensitivity to electromagnetic fields seem to be particularly worth of attention. The idea that some individuals are more sensitive to the electromagnetic field than others, due to genetic background or/and current health status, appears very attractive and should be a subject of further studies. It is worth to note that inconsistent results have been also obtained in the studies dealing with other effects of electrical, magnetic, and electromagnetic fields on organism, including their tumor-promoting action [153–157].

Despite divergences in the reported results, ELF-MF and radio frequency electromagnetic field have to be considered as factors possibly influencing the circadian system function, because a substantial number of studies demonstrated the changes in melatonin and cortisol secretion as well as in sleep after exposition to these fields. Due to widespread exposure of humans and animals to ELF-MF and radio frequency electromagnetic field, the studies on their biological effects should be continued. An important and still unsolved issue is relationships between physical characteristics and biological effects of the fields as well as the mechanisms of field action on the circadian system.

In light of the existing literature, the hypothesis pointing to the disruption of melatonin secretion, as one of the main factors responsible for cancerogenic effects of electrical, magnetic, or electromagnetic fields [158, 159], is not supported by the epidemiological and experimental data. Therefore, it should be currently considered as negatively verified.

## Figures and Tables

**Table 1:** A list of various sources of electromagnetic fields/radiation influencing living organisms [7].

Level	Frequency range	Radiation source
Static	0 Hz	Earth, video screens, magnetic resonance imaging, and other diagnostic/scientific equipment, electrolysis, welding

Extremely low frequency fields	0–300 Hz	Power transmission lines, home wiring, car electric engines, electric trains and trams, welding devices

Intermediate frequency	300 Hz–100 kHz	Video screens, antitheft devices used in cars, homes, shops, card readers, metal detectors, magnetic resonance imaging, welding devices

Radio frequency	100 kHz–300 GHz	Radio, television, mobile phones, microwave ovens, radar and radio transmitters, magnetic resonance imaging
